# Energy-Efficient, Cluster-Based Routing Protocol for Wireless Sensor Networks Using Fuzzy Logic and Quantum Annealing Algorithm

**DOI:** 10.3390/s24134105

**Published:** 2024-06-24

**Authors:** Hongzhi Wang, Ke Liu, Chuhang Wang, Huangshui Hu

**Affiliations:** 1 College of Computer Science and Engineering, Changchun University of Technology, Changchun 130012, China; wanghongzhi@ccut.edu.cn (H.W.); liuke0809@163.com (K.L.); huhuangshui@ccut.edu.cn (H.H.); 2 College of Computer Science and Technology, Changchun Normal University, Changchun 130032, China

**Keywords:** wireless sensor networks (WSNs), clustering, fuzzy logic, routing, quantum annealing algorithm

## Abstract

The main limitation of wireless sensor networks (WSNs) lies in their reliance on battery power. Therefore, the primary focus of the current research is to determine how to transmit data in a rational and efficient way while simultaneously extending the network’s lifespan. In this paper, a hybrid of a fuzzy logic system and a quantum annealing algorithm-based clustering and routing protocol (FQA) is proposed to improve the stability of the network and minimize energy consumption. The protocol uses a fuzzy inference system (FIS) to select appropriate cluster heads (CHs). In the routing phase, we used the quantum annealing algorithm to select the optimal route from the CHs and the base station (BS). Furthermore, we defined an energy threshold to filter candidate CHs in order to save computation time. Unlike with periodic clustering, we adopted an on-demand re-clustering mechanism to perform global maintenance of the network, thereby effectively reducing the computation and overhead. The FQA was compared with FRNSEER, BOA-ACO, OAFS-IMFO, and FC-RBAT in different scenarios from the perspective of energy consumption, alive nodes, network lifetime, and throughput. According to the simulation results, the FQA outperformed all the other methods in all scenarios.

## 1. Introduction

Data transmission, as the core element in information and communication technology, is a crucial component of modern communication networks. Efficient methods for data acquisition and transmission paths have theoretical and practical implications in the application of WSNs that can effectively save energy, thus extending the network lifetime and improving network feasibility for real-time applications [1,2]. Sensor nodes, which are distributed across a wide area, have the capability to acquire information at any time, in any place, and in any environment. As a result, WSNs find wide applications in industry [3], transportation [4], medical care [5], space exploration [6], and other fields that serve as the foundation for the development of the Internet of Things (IoT) [7,8]. Nevertheless, most sensor nodes have limited energy, computing, storage, and communication capabilities because the batteries they are equipped with are almost impossible to replace or continuously charge at any time [9]. Moreover, the continuous and independent data transmission between nodes and other nodes and between nodes and the base station increases the loss of power and hastens node mortality. Therefore, scholars have focused on how to effectively and energy-efficiently utilize sensor nodes for data acquisition and transmission, as well as analysis and processing via upper-level applications and users [10]. In order to realize this goal, many strategies have been proposed, with clustering and routing protocols being identified as the most effective ways [11].

A clustering algorithm divides sensor nodes into several independent groups, referred to as clusters [12]. Each cluster consists of a cluster head (CH) node and several ordinary nodes. Intra-cluster and inter-cluster communications lower the cost of the distance between the farthest nodes and the sink, and using data aggregation to transmit information consumes significantly less energy. In general, sensor nodes are arbitrarily distributed in the network; hence, the focus of research is on allocating nodes to the nearest designated CH. Unreasonable allocation will result in an unequal number of sensor nodes, resulting in uneven residual energy. Fuzzy logic performs well in optimization decision making [13,14,15], which is being used to resolve uncertainties in CH selection based on predefined fuzzy rules. Unlike the conventional technique, each variable in fuzzy logic is assigned a value between 0 and 1, signifying its correctness or incorrectness [16]. Fuzzy clustering algorithms typically set fuzzy rules based on input variables such as the residual energy of nodes, the distance to nodes, or the distance to the base station (BS) as input variables. Then, candidate nodes with the highest fuzzy outputs are selected as cluster heads (CHs), resulting in the appropriate distribution of CHs in a network [17]. Fuzzy logic has almost the same structure as human logic, and it can adapt to changing environments well, according to preset rules. Most protocols modify the node energy in each round, and CHs are adjusted based on fuzzy rules that can greatly prolong the network’s lifetime. However, in large networks, complex fuzzy rules demand a lot of computational resources and time.

The potential parallelism and distributed characteristics of meta-heuristic algorithms can effectively solve complex nonlinear problems, and their global optimization capability is ideal for solving NP-hard problems in wireless sensor networks that can achieve outcomes with fewer parameters. A meta-heuristic algorithm converts the selection of CHs and a routing path into an optimization problem with a specific objective function. Then, it continuously iterates until the optimal solution of the function is found. For example, the cat swarm algorithm [18], fish migration optimization algorithm [19], ant colony optimization algorithm [20], and genetic algorithm [21] are used to select CHs and find routing paths in a clustering and routing protocol. The calculation results of most meta-heuristic algorithms are random due to the initial parameter settings, and there is a risk of falling into a local optimum when dealing with complex problems. On this basis, the combination of various meta-heuristic approaches is derived to solve multi-objective problems, such as butterfly algorithm-based clustering combined with ant colony optimization-based routing [22], improved duck and traveler optimization-based clustering combined with artificial gorilla troop optimization-based routing [23], hybrid gray wolf optimization, and the marine predator algorithm for clustering integrated with hybrid gray wolf optimization and the graph model for routing [24]. However, the complexity of the protocols is largely increased for execution. Therefore, hybrid meta-heuristic and traditional protocols are utilized to balance the overall performance of a network. For instance, the BAT algorithm for clustering combined with the fuzzy logic-based routing technique [14] adjusts the control parameters of fuzzy rules with the shuffled frog-leaping algorithm [17], and fuzzy logic model-based clustering was combined with particle swarm optimization-based routing [25].

In recent years, the universal applicability of quantum mechanics has been continuously proved, and the integration of quantum theory into intelligent optimization has emerged as an attractive research branch. Due to its ability to significantly enhance the solution speed compared to classical algorithms, scholars have incorporated concepts and mathematical mechanisms from quantum computing into existing optimization algorithms. For instance, they have integrated the concept of quantum computing into the traditional tunicate swarm algorithm to solve objective functions and obtained a clustering scheme that minimizes network energy consumption [26]. Shakir Mahoomed Abas et al. combined quantum coding with a genetic algorithm and used qubits to represent candidate routes that explore and evaluate multiple routes simultaneously through quantum parallelism [27]. As a classical meta-heuristic algorithm, the simulated annealing (SA) algorithm is widely used in wireless sensor routing protocols. Despite being a random-step process, the algorithm is prone to stay in a local optimum when the objective function has many high obstacles [28]. To overcome this problem, the quantum annealing (QA) algorithm was developed by replacing the temperature parameter of the SA algorithm with the intensity of the tunneling field. The quantum wave motion is generated through the mechanism of quantum tunneling, enabling the attainment of an optimal point across the potential barrier [29]. WSNs consist of a multitude of nodes and complex network topologies, and the network environment changes constantly over time. Consequently, it is imperative for algorithms to possess a certain level of adaptability. The quantum annealing algorithm demonstrates robust global search capability, effectively optimizing routing schemes. Its inherent parallelism enables efficient handling of large-scale problems and facilitates rapid convergence speed, thereby significantly enhancing routing efficiency and prolonging network lifespan. We utilized the accuracy of the fuzzy logic in optimization decisions to select CHs. Considering that Type-2 fuzzy rules require a large amount of computing time, we adopt the quantum annealing algorithm with a fast convergence rate in the routing stage. The proposed protocol in this paper aims to transmit data efficiently and effectively while also conserving energy and reducing computational time.

In this paper, a hybrid of a fuzzy logic system and a quantum annealing algorithm-based clustering and routing protocol (FQA) is proposed to improve the stability of the network and extend the network lifetime. The protocol selected CHs from all candidate nodes with higher residual energy rates, more neighbors, shorter distances to the BS, and shorter average distances to the member nodes than other candidate nodes and planned the most energy-efficient routing path from the CHs to the BS. Furthermore, the protocol adopts an on-demand re-clustering mechanism instead of clustering periodically or round-by-round like other protocols. The proposed protocol was compared with other latest protocols FRNSEER, FC-RBAT, BOA-ACO, and OAFS-IMFO in four scenarios. The simulation results showed that our protocol is significantly better than other protocols in terms of alive nodes, network lifetime, energy consumption, and throughput. The main contributions of this paper are summarized as follows:

- We utilized fuzzy logic-based clustering and quantum annealing algorithm-based routing to establish an energy-efficient data transmission mode that can effectively prolong the network lifetime;
- We adopted an energy threshold to filter candidate CHs to save computation time;
- Application-based FIS is used to select appropriate CHs from candidate CHs and considers four parameters as fuzzy input variables: residual energy, the number of neighboring nodes, distance to the BS, node centrality;
- A two-dimensional routing matrix and weight matrix are constructed to obtain the objective function. Moreover, we used the quantum annealing algorithm to evaluate the objective function and then obtain the optimal routing route from CHs to the BS;
- An on-demand re-clustering mechanism is used to maintain clusters locally and globally for computation and message overhead reduction.

The remaining parts of the paper are organized as follows: Section 2 reviews the related literature. Section 3 introduces the system model in detail. Section 4 describes the proposed protocol FQA in detail. Section 5 discusses the performance of the proposed methodology with the existing protocols. Finally, Section 6 presents the conclusion.

## 2. Related Works

Traditional clustering usually refers to hard clustering in which each node is classified into a unique category, clusters are independent of each other, and nodes belong exclusively to one cluster. Node grouping can be based on different parameters, mainly residual energy and physical proximity [12]. For instance, K-means clustering is an iterative distance-based clustering algorithm that updates CHs based on the distance of nodes to the cluster center [30]. In view of the problems existing in classical clustering and routing protocols, such as CH selection, difficulty in handling large-scale data, and incomplete parameter selection, scholars have been trying to improve traditional algorithms and propose new algorithms to solve the above shortcomings and improve the performance of the protocol.

The exceptional multi-objective optimization capabilities of meta-heuristic algorithms have enabled them to be extensively employed by scholars in WSNs. In [18], CSO is used to optimize the selection of CHs by simulating the tracking mode of a cat. The received signal strength, residual energy, and intra-cluster distance are considered factors in this scenario, and only CHs are allowed to communicate with the BS. The authors completed indoor experiments with sensor nodes, a microcontroller, and a LabVIEW interface. The results showed that the algorithm has a significant improvement in extending the network lifetime and reducing the computation time. However, the performance of the network is affected when the cluster has numerous nodes. In [22], the BOA (butterfly optimization) algorithm is proposed to optimize the selection of CHs, constructing an objective function based on factors such as residual energy, distance to neighbors, distance to the BS, node degree, and node centrality. Then the ACO (ant colony optimization) algorithm is adopted to plan the optimal transmission path from the source node to the BS via CH. The distance to the BS, residual energy, and node degree are taken into consideration. Compared with traditional algorithms and some existing methods, the network lifetime is significantly improved. In [24], a two-tier protocol for clustering and routing (THSI-RP) was proposed. In the first tier, THSI-RP designed the objective function incorporating energy, the intra-cluster distance, the distance between CH and BS, and node centrality as parameters to select the optimal CHs. Additionally, GWO (grey wolf optimization) and MPA (marine predator algorithm) were combined for clustering to enhance search efficiency. In the second tier, THSI-RP established a communication routing tree between CHs and BS to achieve the shortest data transmission path. Experimental results showed that THSI-RP can effectually improve the network lifetime, but a two-tier protocol design results in a higher network complexity. The two-level genetic algorithm [31] introduced a novel approach to solve two problems simultaneously: selection of CHs and optimal multi-hop routing. In this proposed method, the energy consumption of the multi-hop routing, designed by the second-level algorithm, is considered as a parameter when evaluating the chromosomes in the first-level algorithm. The experimental results showed that network lifetime has been increased when compared to three approaches. The selection of CHs and the routing among them are performed separately. Furthermore, the GA algorithm was substituted with another meta-heuristic algorithm for a two-level framework, and the results of the two methods are quite close. It can be concluded that the results obtained from the proposed framework are not heavily influenced by the algorithms used for optimization. In [32], S. Jagadeesh et al. proposed a novel protocol for clustering and routing. OAFS-IMFO used the OAFS (oppositional artificial fish swarm) algorithm to optimize the selection of CHs, which constructed an objective function based on residual energy, distance between CHs, and distance between CHs and CMs (cluster members). Subsequently, the IMFO (improved moth flame optimization) algorithm was adopted to plan the shortest transmission path from CHs to the BS, including factors such as residual energy of CHs and distance among CH, relay CH, and BS. Compared with some existing protocols, OAFS-IMFO achieves significantly higher packet throughput while consuming more energy.

The proposed hybrid differential evolution and simulated annealing (DESA) algorithm in [33] aims at maximizing the network lifetime of the WSN by combining the simulated annealing (SA) approach for global optimal solution with the DE approach for local search. The DESA algorithm takes only two factors into account, energy and distance between nodes, in the process of selecting cluster heads, which is not comprehensive enough. As a classic meta-heuristic algorithm, the SA algorithm has been widely applied in WSNs. However, it still has several limitations, such as easily falling into local optima. With the rapid development of quantum technology, scholars have integrated the concept and mathematical mechanism of quantum computing into existing optimization algorithms to improve their ability to search the solution space. Among these approaches, the quantum annealing algorithm swiftly emerged as a classical discrete quantum technology in quantum optimization algorithms. Compared to the simulated annealing algorithm, it can solve the NP-hard problem in WSNs more effectively. In [34], the clustering objective function was reformulated as a quadratic unconstrained binary optimization (QUBO) problem, and the paper proposed two encoding methods. One is the one-hot encoding, which associates each data point with a k-bit string, but this method fails to utilize quantum bits effectively and has inherent limitations; the other is binary encoding, which represents each data point’s classification by a binary string and is executed hierarchically. The proposed method needs to be implemented with the help of commercially available quantum annealing hardware and a purely classical solver, “qbsolv” released by D-Wave. In [29], the mobile sink node collects data from all nodes within the communication range along the planned path and returns to the initial location after completing one round. The problem of finding the shortest path for a mobile sink node is essentially a traveling salesman problem (TSP). Zhijie Huang et al. employed quantum encoding techniques to represent the mobile path and utilized the Hamiltonian of the QA algorithm to guide the operation of a quantum revolving gate which effectively addresses the energy hole problem.

With the excellent performance of fuzzy theory in optimizing decisions and reducing energy consumption in WSNs, fuzzy logic is widely used in selecting CHs and clustering. Utilizing the fuzzy logic control system enables real-time adjustments based on dynamic environmental conditions, thereby enhancing the fault tolerance and stability of the system. Combining fuzzy logic and meta-heuristic algorithms can fully leverage their respective strengths and enhance capabilities in addressing intricate optimization problems. The generation of fuzzy rules in [13] involves considering energy and utility factors, and the fuzzy inference system is employed to identify relay nodes. The minimum length route is determined based on coverage distance, energy, utility factor, and bandwidth availability. Amruta Lipare et al. [14] proposed load-balanced fuzzy C-means (FCM) algorithm-based clustering integrated with BAT approach-based routing (FC-RBAT). They used FCM to form clusters and find the centroid of the clusters. Then, they selected proper CHs among the sensor nodes from each cluster, which constructed the objective function based on residual energy, intra-cluster distance, distance to cluster centroid, and distance to the BS. The routing process is conducted using a BAT-inspired approach that considers the lifetime of CHs, the number of hops, and total transmission distance as essential parameters. In [17], the suggested protocol used the shuffling frog-leaping algorithm (SFLA) to pick relay nodes and CHs by optimizing two fuzzy rule tables. Firstly, FMSFLA designed fuzzy rules based on the parameters residual energy, distance to the BS, and the number of neighboring nodes to choose candidate CHs and ensure an appropriate distribution of CHs by specifying the overlap threshold between CHs. In the second phase, FMSFLA selected the relay nodes named parents from the candidate nodes based on the following parameters: energy, distance between each node, and the mean load of the selected route between the candidate CH node n and the BS. Simulation results demonstrated that FMSFLA can achieve a consistent network workload, lower energy consumption, and extend the network lifetime, but it requires off-line optimization. Yanika Kongsorot et al. [35] developed a fuzzy-based protocol by using an updated SFLA algorithm. They designed a new encoding scheme that utilized three fuzzy inference rules (FIS) to compute the odds of becoming a CH, the specific CH of a non-CH, and identifying an NH (next-hop node). EFC-ISFLA introduced the historical selection of nodes as CHs and defined an overlay boundary that sets the minimum allowable distance between adjacent CHs. In contrast to previous research [17], the improved SFLA (ISFLA) combined multiple OBL strategies to enhance the synergy between search algorithm advancements and exploratory capabilities. The comparison results showed that the protocol outperformed its competitors in terms of network stability, network lifetime, data transmission, and the correctness of test functions. Nevertheless, the superior performance of the protocol is accompanied by a significant increase in computation time, a more enormous load on the base station, and an increase in cost. In [36], the proposed designed Type-2 fuzzy logic system for CH selection was combined with a BFO (bacterial foraging optimization) routing algorithm. The node degree, node centrality, and energy are utilized as three fuzzy inputs to calculate the node’s probability of becoming CH. However, the generated 27 rules still exhibit low accuracy in solving the objective function.

## 3. System Model

### 3.1. Network Model

**Figure 1:**
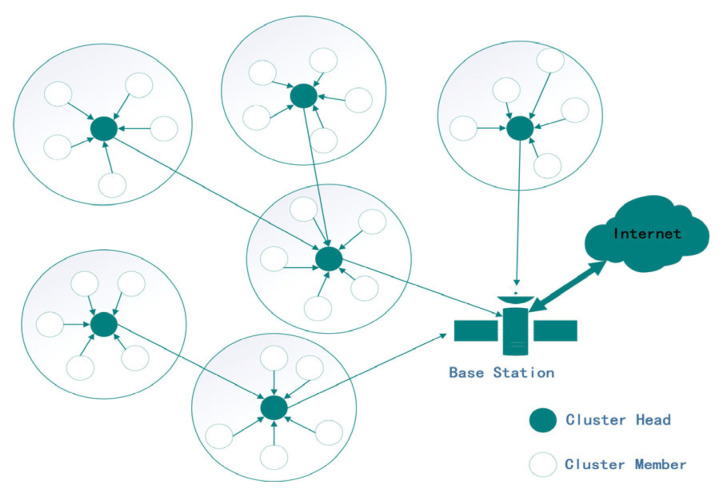
WSN network model.

Assuming that N nodes are randomly dispersed within a Z*Z two-dimensional square, the CHs collect sensed data from all CMs (cluster members) through clusters, aggregate them, and then transmit data to the BS. The structure of WSN is depicted in Figure 1.

The network model is formulated based on the following considerations:

- The sensor nodes are homogeneous and static and are identified by their corresponding IDs;
- The distance between nodes is estimated based on the received signal strength indicator (RSSI);
- Wireless connections are bidirectional and symmetric;
- No energy constraint is attached to the BS;
- The sensor nodes communicate their information to the CHs and are in charge of collecting, aggregating, and sending the information to the BS directly or through other CHs.

### 3.2. Energy Model

The first-order radio model refers to the classic Leach protocol and the literature [16,30] to calculate the dissipated energy in this paper. The energy consumption for transmitting between nodes i and j with bits of data over distance d is as follows:

(1) $$E_{TX(i,j)}=\left\{\begin{array}{c} k*E_{elec}+k*\varepsilon_{fs}*d^{{\;\;}^{2}},d<d_{0}\\ k*E_{elec}+k*\varepsilon_{mp}*d^{4},d>d_{0} \end{array}\right.$$

where $E_{elec}$ is the energy consumed in a transmitter or receiver circuit to send or receive one bit of packet data, and $d_{0}$ is the threshold distance of the transmission information which is estimated by $d_{0}=\sqrt{\varepsilon_{fs}/\varepsilon_{mp}}$. $\varepsilon_{fs}$ and $\varepsilon_{mp}$ are the amplifier coefficients used for free space and multi-path models. Similarly, the energy consumption for receiving between nodes i and j with k bits of data can be calculated according to Equation (2):

(2) $$E_{RX(i,j)}=k*E_{elec}$$

Furthermore, the energy consumed for aggregating k bits of data can be estimated by

(3) $$E_{DA}=k*E_{pdb}$$

where $E_{pdb}$ is the energy consumed for fusing 1-bit data.

## 4. Proposed Protocol

The FQA is a centralized protocol, and all computations are executed within a processor located in the BS. The objective is to develop an energy-efficient clustering and routing protocol that should be adjustable. We have divided the solution into four phases: CH selection, cluster formation, multi-hop routing, and on-demand re-clustering. The details of each phase are elaborated as follows.

### 4.1. CH Selection

The flexibility and fault tolerance of fuzzy logic enable simultaneous consideration of multiple factors when selecting the appropriate CH. The applicability of CHs can be evaluated comprehensively by defining appropriate membership functions and fuzzy rules. In the proposed FQA, we utilized an FIS (fuzzy inference system) with the classic Mamdani model to select the appropriate CHs based on the well-tuned fuzzy rule. In each round, the BS collects the information of all nodes in the network to select the set of CHs.

#### 4.1.1. Step 1: Selection of Candidate CHs

Because nodes cannot be charged and their energy is limited, nodes with sufficient energy should be selected as CHs to improve network efficiency. The nodes with energy levels higher than the energy threshold are considered candidate CHs. The set of candidate CHs selected can be mathematically expressed by Equation (4):

(4) $$S_{CH}=\left\{N_{i}\left|E\left(N_{i}\right)\geq E_{threshold}\right.\right\}$$

where $E\left(N_{i}\right)$ is the residual energy of the node i and $E_{threshold}$ represents the average energy of the existing alive nodes:

(5) $$E_{threshold}=\frac{\sum_{i=1}^{N_{alive}}E\left(N_{i}\right)}{N_{alive}}$$

Equation (5) is used to determine the nodes with enough energy to be selected for CHs.

#### 4.1.2. Step 2: Selection of CHs

In this step, an FIS is adopted to compute the probabilities of becoming a CH for all candidate CHs stored in $S_{CH}$. We employed four fuzzy inputs including residual energy, number of neighboring nodes, distance to the BS, and node centrality to maximize the rationalization of CH selection. The parameters considered as FIS input characteristics are detailed as follows:

- Residual energy ($f_{CH_{1}}$): This parameter indicates the remaining energy of node i denoted by $E\left(N_{i}\right)$ in the current round. The CH serves as a critical node for data transmission, requiring a large amount of energy. This characteristic becomes more critical in the later stages of operation. In contrast to existing protocols that use a linearly declining weight constraint on this parameter, our approach determines the energy threshold at step 4.1.1 and selects nodes with the highest residual energy as candidate CHs.
- Number of neighboring nodes ($f_{CH_{2}}$): This parameter refers to the number of direct communication connections established between each node within its communication range and its surrounding nodes, representing each cluster’s compactness. A larger value of this parameter is considered a more satisfactory feature because it indicates a shorter intra-cluster distance.
- Distance to the BS ($f_{CH_{3}}$): This parameter concerns the Euclidean distance between the candidate node i and the BS which is expressed by $Dis\left(i,BS\right)$. The nodes with a closer distance to the BS have a higher probability of being selected as CHs because they consume less communication energy. Since the nodes are static, the BS has collected the distance information of all nodes based on RSSI in the first iteration.
- Node centrality ($f_{CH_{4}}$): Our protocol in this paper selects CHs before cluster formation, so we use the average distance between a candidate CH and its set of neighboring nodes ($Neighbor\left(N_{i}\right)$) [35] to represent node centrality, which can be computed by

(6) $$f_{CH_{4}}=\frac{\sum_{i\in Neighbor\left(N_{i}\right)}d\left(CH,i\right)}{Neighbor\left(N_{i}\right)}$$

where $d\left(CH,i\right)$ is the Euclidean distance between candidate CH and one of its neighbors. The smaller the value of $f_{CH_{4}}$, the closer the candidate CH is to the center of the cluster. Nodes selected as CHs that are close to the cluster centroid can minimize the energy consumption of data transmission.

**Figure 2:**
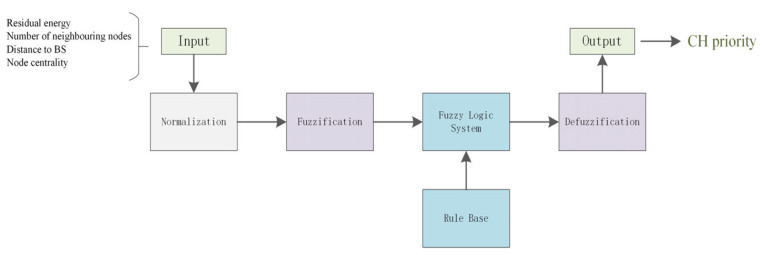
FIS for FQA.

Using the above-mentioned inputs, the FLS outputs a chance to indicate the priority of nodes becoming CHs. The whole process involves normalization, fuzzification, a fuzzy logic system, and defuzzification, as shown in Figure 2.

Where normalization aims to standardize input variables from different ranges into a uniform range between 0 and 1, this step maps membership values to a standardized range that allows us to make efficient comparisons and calculations. Each normalized variable is mapped to a corresponding fuzzy linguistic variable via the membership functions.

In our proposed method, each selected input variable of each fuzzy system has different membership functions represented as trapezoidal, triangular–trapezoidal, and triangular. The fuzzy outputs for all the FISs are derived from the set of selected features obtained by the optimization process. We employed seven linguistic variables to express the outputs, providing enhanced flexibility in formulating the fuzzy rules. The detailed linguistic variables and parameters can be found in Table 1.

**Figure 3:**
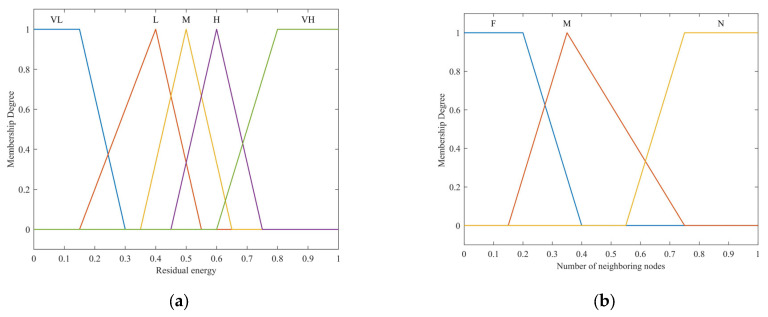

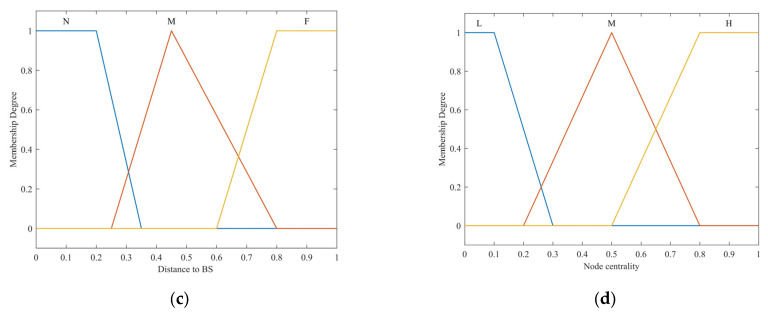
Membership function for the input variables: (**a**) residual energy; (**b**) number of neighboring nodes; (**c**) distance to the BS; (**d**) node centrality.

**Figure 4:**
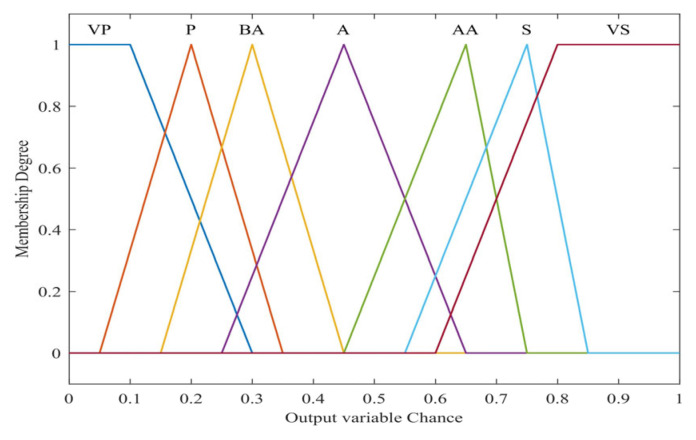
Membership function for the output variable.

The membership functions of the inputs and output can be formulated based on previous works [13,17] and experimental experiences. These are illustrated in Figure 3 and Figure 4, respectively.

In the proposed FQA, we used the fuzzy logic model to select CHs based on probability values. The node with the highest probability values emerges as the winner and takes the CH position. Every node sends messages containing the node ID and probability value to all other nodes in the range after the fuzzy system generates the probability values. They receive a message with the node ID and probability from other nodes and use this information to select a CH. The function mapping between input and output variables is constructed based on the knowledge base consisting of if–then rules, and the final fuzzy rules are shown in Table 2.

### 4.2. Cluster Formation

After the selection of the CHs, each final selected CH broadcasts the message that it becomes a CH within its communication range. The nodes that are not selected are treated as CMs (cluster members) and assigned to a particular CH with higher potential according to Equation (7):

(7) $$N_{p}=\frac{\alpha E_{ch}\left(i\right)}{Dis\left(S_{j},CH_{i}\right)}$$

where $N_{p}$ is the node potential, $E_{ch}\left(i\right)$ denotes the residual energy of the respective CH, and $Dis\left(S_{J},CH_{i}\right)$ represents the Euclidean distance between the $CH_{i}$ and $S_{j}$. α is the proportionality constant. Since the node with higher residual energy has been selected as the CH in step 4.1.1, $Dis\left(S_{J},CH_{i}\right)$ is regarded as a more important factor in this section. After a simulation comparison, we set α to 0.3.

### 4.3. Multi-Hop Routing Approaches

#### 4.3.1. Simulated Annealing

Inspired by the physical annealing process, Metropolis et al. simulated the annealing process in metal smelting and proposed the simulated annealing algorithm, which is a Markov chain Monte Carlo method based on statistical mechanics, to find the optimal solution that minimizes the objective function among a set of candidate solutions. At the beginning of the annealing process, the SA algorithm avoids becoming stuck in local optima by occasionally moving uphill, which iteratively compares the value of the objective function corresponding to the current point and the adjacent points. In a later stage, it incorporates the Metropolis criterion to find the minimum value of the objective function. Although the SA algorithm is widely used in path planning, it still has many shortcomings. Parameters such as initial temperature and iteration times of the inner loop have a great influence on the results, and at the beginning of annealing, the probability of accepting the better solution and the worse solution is almost the same.

#### 4.3.2. Quantum Annealing

**Figure 5:**
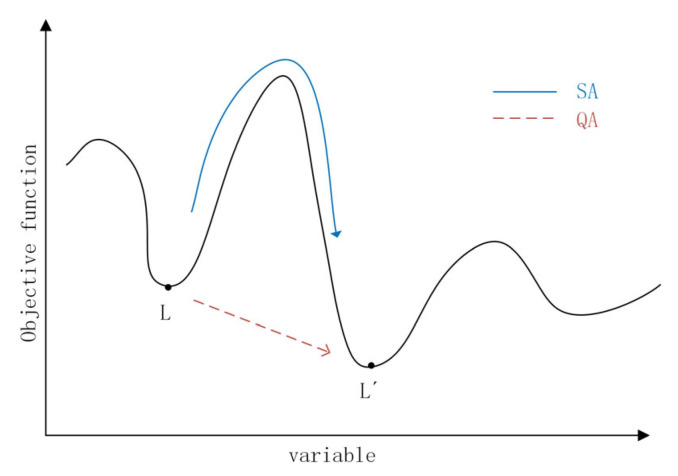
Difference between SA and QA.

Different from the simulated annealing algorithm which jumps over the barrier to eliminate the local optimum, the quantum annealing algorithm introduces a penetration field into the quantum system and generates quantum wave motion to penetrate the potential barrier itself, then reaches the lowest energy state [29]. In the early stage of annealing, the kinetic energy term is relatively large, providing a large disturbance that can fully traverse the entire solution space. The kinetic energy term is gradually reduced, and in the later stage of annealing, the kinetic energy term is gradually reduced to 0. The comparison of the ways of crossing the barrier is shown in Figure 5.

**Figure 6:**
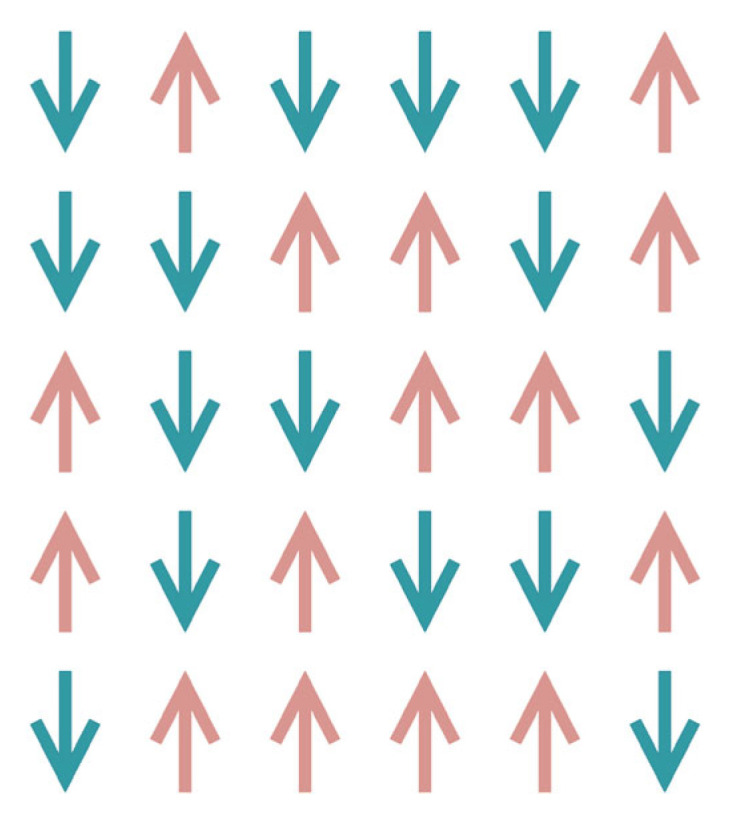
Two-dimensional stochastic Ising model.

In other words, the state of the minimum potential energy of the system is reached. The evolution of the quantum system under the action of potential and kinetic energy can be described by the following Schrödinger Equation (8):

(8) $$i\hbar \frac{\partial }{\partial r}\Psi \left(r,t\right)=\hat{H}\Psi \left(r,t\right)$$

where ℏ is Planck’s constant and $\hat{H}$ is the Hamiltonian operator.

(9) $$\hat{H}=-\frac{\hbar^{2}}{2m}\nabla^{2}+V\left(r\right)$$

where m is the particle mass, ∇ is the gradient operator, $V\left(r\right)$ is the potential field of the particle. In practice, the complexity of Equation (9) increases exponentially with the increase in problem complexity, so we adopt the PIMC (path-integral Monte Carlo) method to simulate the quantum annealing process. The two-dimensional stochastic Ising model is employed to elucidate the PIMC process [37]:

As shown in Figure 6, each arrow $a_{i}\in \left\{+1,-1\right\}$ represents a distinct magnetic needle with varying spin magnetism. The collective spin orientation of all these magnetic needles determines the overall magnetization. The interaction between adjacent magnetic needles and environmental thermal noise influences the behavior of each needle. Thermal noise interference intensifies as temperature increases, leading to random magnetism in the magnetic needles. However, during the annealing process characterized by a gradual decrease in temperature, neighbor interactions become more prominent. Eventually, when the temperature reaches a sufficiently low level, many tiny magnetic needles in a uniform direction exhibit magnetism. At this time, the system energy is minimal. The Hamiltonian function of the transverse field Ising model is formulated as

(10) $$H_{p}\left(t\right)=\sum_{i=1}^{N}h_{i}\sigma_{i}{\;\;}^{Z}+\sum_{i,j=1}^{N}J_{i,j}\sigma_{i}{\;\;}^{Z}\sigma_{j}{\;\;}^{Z}$$

where $h_{i}$ represents the external local field, which is not considered in this paper for the sake of simplicity and is assumed to be 0. $J_{i,j}$ denotes the coupling strength between quantum bit i, j; $\sigma_{i}{\;\;}^{Z}$ and $\sigma_{j}{\;\;}^{Z}$ denote the Pauli operator Z spin matrices at point i, j. The concept of spin can be interpreted as a minute magnetic needle positioned in a specific state, oscillating between the states of 1 and 0 due to the influence of the transverse field’s kinetic energy. This transformation effectively shifts the model from classical to quantum. According to the Hamiltonian function of the Ising model, the Hamiltonian function of quantum annealing can be obtained:

(11) $$H(t)=H_{p}\left(t\right)+\Gamma (t)\sum_{i,j=1}^{N}\Delta_{i}\sigma_{i}{\;\;}^{x}$$

where Γ stands for field strength, and it induces the up-and-down transition of the spin state of a single quantum bit, similar to the temperature T in the simulated annealing algorithm. According to the Trotter theorem, we decompose the Hamiltonian into multiple smaller Hamiltonians to efficiently compute the evolutionary outcomes.

#### 4.3.3. Route Generation

A. The basic process of the QA algorithm

The limitation of the uncertain convergence time is overcome by optimizing the QA algorithm. The route generation process using the QA algorithm is described clearly in this section.

1. The BS collects the location information and the residual energy information of each CH.
2. We constructed a two-dimensional routing matrix L and a two-dimensional weight matrix ω according to the number of CHs and energy consumption model.
3. In order to minimize the energy consumption in the communication process, the objective function is constructed by using two matrices.
4. We constructed constraint terms and combined them with the objective function to obtain the potential energy.
5. The kinetic energy of the construction is combined with the potential energy to obtain the Hamiltonian.
6. The routing planning scheme is obtained by solving the resulting Hamiltonian with the QA algorithm and repeating N times. Then, the scheme with the minimum energy consumption is taken as the current round scheme.
7. The energy consumption difference between the current and last rounds was compared. If the relative difference was within 5%, the scheme with more minor energy consumption was selected as the optimal scheme; otherwise, the next round of comparison was performed.

B. Construction of objective function and potential energy term

The matrix L which represents the routing connection relationships of all CHs in the network is expressed in Equation (12):

(12) $$L=\left[\begin{array}{ccccccc} L_{1,1} & L_{1,2} & \cdots & L_{1,b} & \cdots & L_{1,C} & L_{1,BS}\\ L_{2,1} & L_{2,2} & \cdots & L_{2,b} & \cdots & L_{2,C} & L_{2,BS}\\ \vdots & \vdots & \ddots & \vdots & \ddots & \vdots & \vdots \\ L_{a,1} & L_{a,2} & \cdots & L_{a,b} & \cdots & L_{a,C} & L_{a,BS}\\ \vdots & \vdots & \ddots & \vdots & \ddots & \vdots & \vdots \\ L_{C,1} & L_{C,2} & \cdots & L_{C,b} & \cdots & L_{C,C} & L_{C,BS} \end{array}\right]$$

where C is the number of CHs. The CHs collect data and transmit them to the BS through multi-hop or single-hop routing, and the BS is the end of the data transmission. $L_{a,b}=0$ means that there is no routing connection between nodes a and b; $L_{a,b}=1$ means that there is a routing connection between nodes a and b. At the same time, we use the energy consumption of single-bit data transmission as the connection weight and only consider the energy consumption of sending data from CHs to the BS when constructing the energy consumption weight matrix in this paper. The two-dimensional weight matrix ω representing the routing energy consumption is as follows:

(13) $$\omega =\left[\begin{array}{ccccccc} \omega_{1,1} & \omega_{1,2} & \cdots & \omega_{1,b} & \cdots & \omega_{1,C} & \omega_{1,BS}\\ \omega_{2,1} & \omega_{2,2} & \cdots & \omega_{2,b} & \cdots & \omega_{2,C} & \omega_{2,BS}\\ \vdots & \vdots & \ddots & \vdots & \ddots & \vdots & \vdots \\ \omega_{a,1} & \omega_{a,2} & \cdots & \omega_{a,b} & \cdots & \omega_{a,C} & \omega_{a,BS}\\ \vdots & \vdots & \ddots & \vdots & \ddots & \vdots & \vdots \\ \omega_{C,1} & \omega_{C,2} & \cdots & \omega_{C,b} & \cdots & \omega_{C,C} & \omega_{C,BS} \end{array}\right]$$

So we obtain the objective function $f_{route}$ in Equation (14), and our optimization objective is to obtain the minimum value of $f_{route}$,

(14) $$f_{route}=\sum_{\left(a,b\right)}L_{a,b}*\omega_{a,b}$$

$L_{a,b}$ represents the routing connection state, corresponding to $a_{i}\in \left\{+1,-1\right\}$ in the Ising model. In order to prevent the possible matrix L from being in an arrangement that is not in line with the actual situation, we set the constraint term P. Since the communication between nodes is bidirectional, it is an equal-probability event that node a transmits data to node b and node b transmits data to the node a, and the randomly generated routing connection easily causes loop breakage. Therefore, the concept of parent node and child node is used to eliminate the error situation. $L_{a,b}=1$ and $L_{b,a}=1$ represent two different connection relationships which cannot both be 1. $L_{a,b}=1$ means that node a is the parent node of node b, and data are transmitted from a to b. We set the first constraint $P_{1}$ that each node can have only one child node, that is, every column has only one element of 1, in which case $P_{1}$ = 0:

(15) $$P_{1}=\sum_{b=1}^{C}\left(\sum_{a=1}^{C}L_{a,b}=1\right)$$

The second constraint term ensures that CHs are connected to a path:

(16) $$P_{2}={\left(Num-1\right)}^{2}$$

We used an array Num to denote the total count of CHs in the matrix L which satisfies the condition of the number of child nodes being zero. If Num=1, that is, only the BS has no child nodes, $P_{2}=0$. Otherwise, $P_{2}>0$, which is an unreasonable routing scheme. The potential energy is calculated from the above objective function and two constraint terms:

(17) $$H_{p}\left(t\right)=f_{route}+\omega_{\max}\left(P_{1}+P_{2}\right)$$

The weight of constraint terms is increased to eliminate incorrect routing schemes. $\omega_{\max}$ is the largest element value in ω; if the routing scheme satisfies the actual situation, $P_{1}=0$ and $P_{2}=0$, that is, the corresponding $H_{p}\left(t\right)$ is smaller.

C. Construction of classical Hamiltonian

The random construction generates n different connection replicas to form the set $M=\left\{L^{1},L^{2},\cdots ,L^{k},\cdots L^{n}\right\}$. The greater the number of copies, the higher the accuracy of the solution; however, this will also lead to an increase in calculation time and cost. After careful consideration, we refer to the parameter value, n = 40, with the best effect after comparative experiments in the literature [38]. Kinetic energy $H_{k}\left(t\right)$ is generated by quantum fluctuations which refer to the interaction between n sets; that is, the values at the same position between adjacent copies are multiplied and summed. We used the scheme in [37] for the calculation:

(18) $$H_{k}\left(t\right)=J_{\Gamma }\left(\sum_{k=1}^{n}\sum_{a,b}L_{a,b}^{k}L_{a,b}^{k+1}\right)$$

where $L_{a,b}^{k+1}=L_{a,b}^{1}$, and $J_{\Gamma }$ is introduced as a coupling term to adjust the magnetic field strength [39], which is calculated by Equation (19):

(19) $$J_{\Gamma }=-\frac{T}{2}\ln\tanh\left(\frac{\Gamma }{T_{q}}\right)>0$$

where $T_{q}=nT$ is the effective QA temperature. As the Monte Carlo step progresses, the tunneling field intensity $\Gamma_{0}$ slowly decreases from a relatively high value. After manual parameter adjustment for testing, the optimal values of $T_{q}$ and $\Gamma_{0}$ were found within the ranges of $T_{q}\in \left(0,1\right]$ and $\Gamma_{0}\in \left(0,3T_{q}\right)$ [40]. According to the Monte Carlo method, the classical Hamiltonian can be expressed as

(20) $$H\left(t\right)=\frac{1}{n}\sum_{k=1}^{n}H_{p}\left(L^{k}\right)-J_{\Gamma }H_{k}\left(t\right)$$

If the number of the same positions with a value of 1 in n sets is higher, there tends to be greater consistency among adjacent copies, and then the value of $H_{k}\left(t\right)$ is higher and the value of $H\left(t\right)$ is lower. The operator set introduced in the subsequent section is utilized for updates, enabling kinetic energy to reach lower values when potential energy cannot be further reduced, thereby escaping local optima. $J_{\Gamma }$ determines the relative importance between the kinetic energy term and the potential energy term. With the annealing process, the sets gradually become similar, $\Delta H_{k}\left(t\right)$ (the difference value between the candidate kinetic energy term and the current kinetic energy term) gradually decreases, and $J_{\Gamma }$, which is inversely proportional to T, gradually increases, ensuring that the kinetic energy term continues to play a role. From the current Hamiltonian, we obtain the optimal two-dimensional routing connection matrix $L_{best}$ using the PIMC-QA algorithm in Matlab.

D. Construction of operator

**Figure 7:**
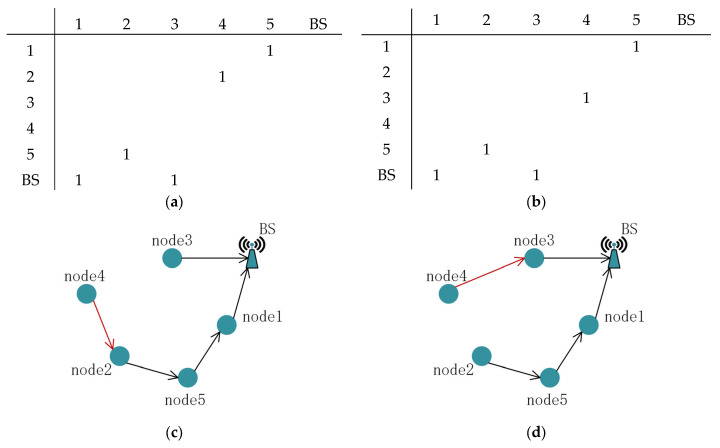
An example of the operator O: (**a**) L matrix before using O; (**b**) L matrix after using O; (**c**) route before using O; (**d**) route after using O.

Given that the constraint term has already been constructed in Section B, we exclusively construct one operation O for updating the replica set. We find a random position in the matrix L with the value of 1, disconnect it from its child node, and then choose a new child node within the same column. Figure 7 shows an example of the operator with arrows indicating the direction of data transmission.

E. The Pseudo-Code of the QA algorithm

Algorithm 1 is the QA algorithm pseudo-code.
**Algorithm 1:** The implementation of route planning$\mathrm{Initialize}:\;\mathrm{replica}\;\mathrm{set}\;M,L_{best}=L^{0},{\Gamma =\Gamma }^{0},M_{c}=Maxsteps,k=0$ $1.\;\mathrm{While}\;\left(\Gamma >0\right)$ $2.\;J_{\Gamma }=-T/2\ln\tan\left({\Gamma /T}_{q}\right)$ $3.\;\mathrm{While}\;\left(k<n\right)$ $4.\;\;L^{k}=O\left\{L^{k}\right\}$ $5.\;\;\Delta H_{p}=H_{p}\left(L^{\prime }\right)-H_{p}\left(L^{K}\right)$ $6.\;\;\Delta H_{k}=H_{k}\left(L^{\prime }\right)-H_{k}\left(L^{k}\right)$ $7.\;\;\Delta H_{C}=\Delta H_{p}/n-J_{\Gamma }\Delta H_{k}$ $8.\;\;\;\;\mathrm{if}\;\left(\Delta H_{p}\right)<=0$ $9.\;\;\;\;L^{k}=L^{\prime }$ $10.\;\;\;\mathrm{if}\;\left(H_{p}\left(L^{k}\right)\right)<=H_{p}\left(\left(L_{best}\right)\right)$ $11.\;\;\;L_{best}=L^{\prime }$ $12.\;\;\;\mathrm{else}\;\mathrm{if}\;\left(\Delta H_{k}\right)<=0$ $13.\;\;\;\;L^{k}=L^{\prime }$ $14.\;\;\;\;\mathrm{else}\;\mathrm{if}\;\left(\exp\left(-\Delta H_{C}/T\right)\right)>random\left(0,1\right)$ $15.\;\;\;\;L^{k}=L^{\prime }$ 16. k=k+1
17.  **end while**

$18.\;T=T-\left(T_{0}/Maxsteps\right),\Gamma =\Gamma -\left(\Gamma_{0}/Maxsteps\right)$

$19.\;M_{c}=M_{c}-1$

20.  **end while**

$21.\;\mathrm{return}\;L_{best}$

22.  **end**

Here, Maxsteps is the maximum number of steps for Monte Carlo steps. $L^{\prime }$ is the candidate solution, which is obtained by updating the current solution with the operator O, and the Monte Carlo criterion [38] of the simulated annealing algorithm is adopted in line 18.

### 4.4. Cluster Maintenance

To reduce energy consumption and balance the load, this paper adopts the on-demand maintenance mechanism. The CHs consume too much energy to collect and transmit data to the BS, and the clusters nearer to the BS consume energy too quickly, so the cluster maintenance phase is considered as one of the important phases for prolonging the network lifetime. Each selected CH remains in its position for a certain number of rounds. Once its remaining energy is below the threshold $\left(E\left(N_{i}\right)<E_{threshold}\right)$, the CH announces the node with the second-largest probability of becoming a new CH. Based on the saved chance values, the CH shift can be performed every time with only a small control packet. Moreover, when a node only functions as a CH in a round, it transmits a small control packet to its neighboring CHs instead of its CMs. Additionally, the data packet will be forwarded by the neighbor CHs until all CHs receive it. The BS will re-initialize the FIS and reselect the CHs on demand when all CHs perform a shift operation or if a CH has exhausted its energy. Then, the quantum annealing algorithm is used to obtain the optimal routing path from CHs to the BS.

## 5. Simulation Results and Discussion

The proposed FQA is simulated using MATLAB 2022a software because its Fuzzy Toolbox examines all fuzzy membership functions, hence making it suitable for use. The performance is compared with the existing protocols, including FRNSEER [13], FC-RBAT [14], BOA-ACO [22], and OAFS-IMFO [32]. The simulation parameters are presented in Table 3.

The positioning of the BS and the number of nodes can influence the experimental outcomes. Thus, we established four different scenarios to evaluate the proposed approach. In scenario 1, we randomly deployed 70 nodes within a network area measuring 100 m × 100 m, and the BS was positioned at the center of the network. In the second scenario, the BS is switched to the edge of the network. Then, we expanded the network to 200 m × 200 m and increased the number of nodes to 150, which can further verify the stability of the proposed algorithm. Finally, we placed the BS at the corner of the network. The scenario parameters are shown in Table 4.

The proposed protocol was compared with the FRNSEER, BOA-ACO, OAFS-IMFO, and FC-RBAT in different scenarios from the perspective of alive nodes, network lifetime, energy consumption, and throughput. The detailed test results are shown and discussed as follows.

### 5.1. Network Lifetime

**Figure 8:**
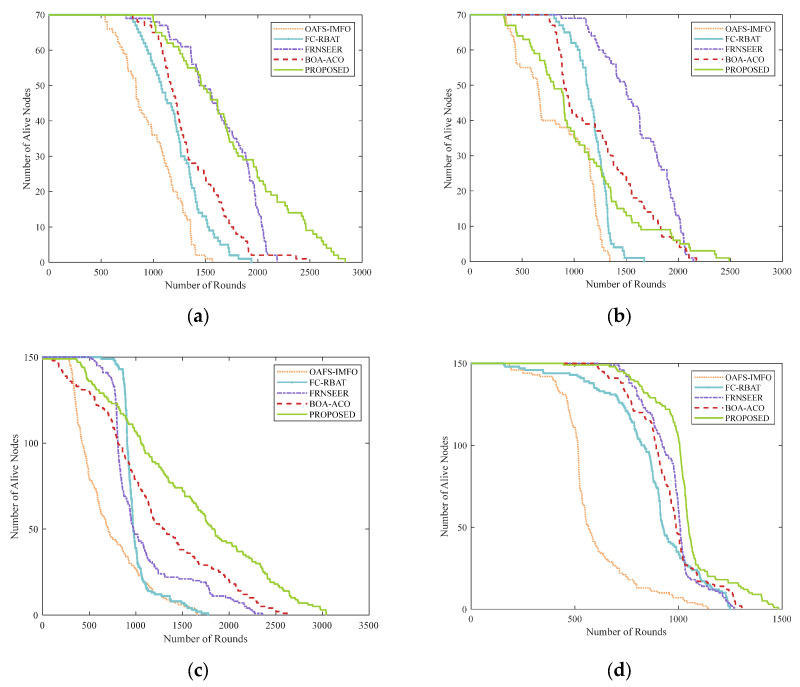
Network lifetime comparison in different scenarios: (**a**) scenario 1; (**b**) scenario 2; (**c**) scenario 3; (**d**) scenario 4.

This section compares the network lifetime between the proposed methodology and existing protocols. The lifetime of a network is defined as the number of rounds when the total number of nodes completely exhausts their energy, and it is the most important metric for measuring the protocol. Table 5 shows the simulation results of the first node death round (FND), half node death round (HND), and all node death round (LND) for different protocols. In addition, the simulation results of the number of surviving nodes in the network are shown in Figure 8.

As shown in Table 5, our proposed protocol overcomes the shortcomings of other protocols while extending the network lifetime; it also performs well in HND and LND. The BOA-ACO protocol utilizes the ACO algorithm to determine the optimal path from CHs to the BS, which has the risk of falling into local optima. OAFS-IMFO introduced two different meta-heuristic algorithms in the CH selection and routing path planning, which increases the risk of falling into the local optimal solution. The two sets of fuzzy rules of FC-RBAT significantly prolong system computation time, leading to higher energy consumption and reduced network lifetime. Our protocol uses the fuzzy system in CHs selection to plan cluster distribution accurately and choose a quantum annealing algorithm in the routing stage. Compared with the FRNSEER protocol, it can quickly obtain the optimal solution in all the transmission paths from CHs to the BS. As shown in Figure 8, our protocol provides better performance in terms of network lifetime. The alive nodes of the proposed protocol are sustained until 2838, 2489, 3040, and 1482 rounds in four scenarios. The performance of the proposed protocol is 81.23%, 85.33%, 83.35%, and 29.66% higher than OAFS-IMFO; 46.36%, 48.77%, 71.27%, and 18.65% higher than FC-RBAT, 29.83%; 16.04%, 28.98%, and 16.60% higher than FRNSEER; and 14.95%, 14.49%, 15.37%, and 13.39% higher than BOA-ACO, respectively, in the different scenarios.

### 5.2. Energy Consumption

**Figure 9:**
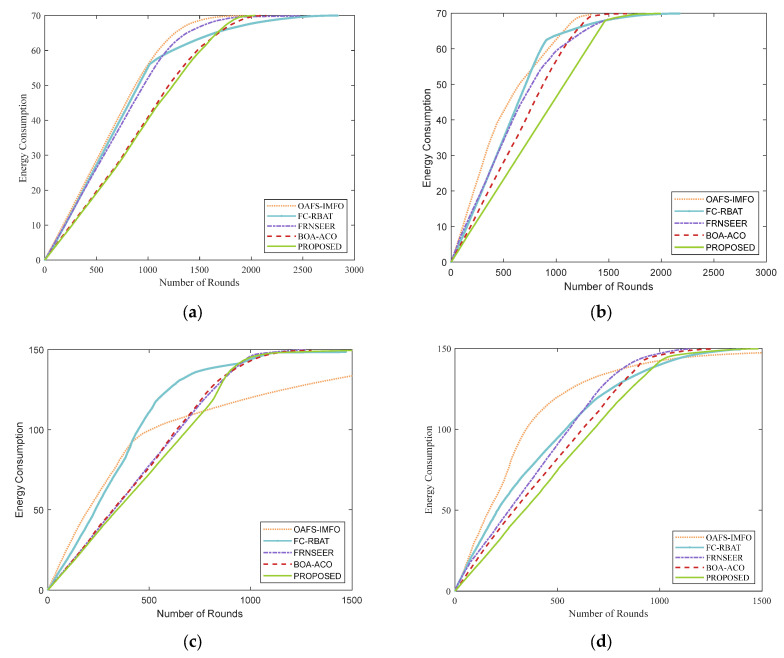
Network energy consumption comparison in different scenarios: (**a**) scenario 1; (**b**) scenario 2; (**c**) scenario 3; (**d**) scenario 4.

The wireless sensor nodes are usually powered by batteries with limited capacity that cannot be easily replaced or recharged promptly. Therefore, effectively reducing energy consumption is crucial for ensuring efficient resource utilization and achieving a sustainable network. Enhancing the system’s sustainability can effectively reduce the frequency and cost of maintenance for networks requiring long-term detection [41]. Figure 9 compares our proposed protocol’s energy consumption with four other latest protocols across four scenarios.

Frequent updates of CHs will increase network energy consumption. Our protocol adds the process of candidate CHs to ensure that the selected CHs are higher than the average remaining energy of existing nodes, prolonging the survival time of CHs and minimizing energy consumption. The FRNSEER and OAFS-IMFO protocols do not consider the degree of cluster compactness, which may cause an uneven distribution of clusters. FC-RBAT used fuzzy C-means for clustering, and its accuracy in selecting reasonable CHs is significantly lower than that of FQA. The parameters considered by the BOA-ACO protocol are more comprehensive, thereby increasing the complexity of calculations. Under identical conditions, our protocol can achieve superior results in terms of clustering and routing.

In the experiment, FQA consumes half of the energy in 880, 752, 518, 503 rounds, which is 43.79%, 96.86%, 58.41%, 93.46% lower than OAFS-IMFO; 37.72%, 49.80%, 49.28%, 40.50% lower than FC-RBAT; 31.54%, 47.75%, 7.25%, 22.68% lower than FRNSEER; and 1.49%, 20.51%, 5.715, 10.55% lower than the BOA-ACO protocol in the four scenarios. FQA has more evident advantages in saving energy.

### 5.3. Throughput

Throughput is a crucial metric for evaluating network performance, as it measures the amount of data that can be successfully transmitted within a given time frame. The results of the simulation comparison demonstrate that our protocol exhibits superior network data transmission efficiency and reliability.

**Figure 10:**
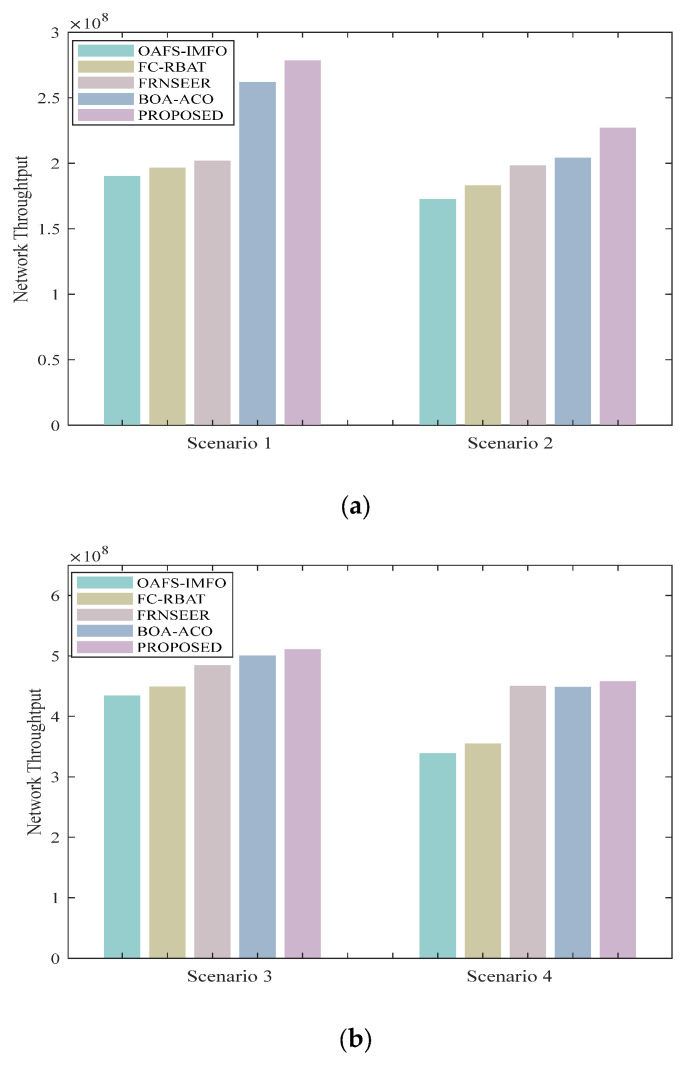
Network throughput comparison in different scenarios: (**a**) area 100 m × 100 m, 15%CH; (**b**) area 200 m × 200 m, 10%CH.

Figure 10 shows the throughput comparison. At the scale of a 100 m × 100 m network, in terms of throughput, compared with OAFS-IMFO, FC-RBAT, FRNSEER, and BOA-ACO, FQA has increased by 38.89%, 32.765%, 26.165%, and 8.72% on average, respectively. These data show that our protocol maintains the effectiveness of data transmission rates as the network expands. FQA has increased by 22.01%, 22,79%, 3.58%, and 2.04% on average at 200 m × 200 m, respectively.

## 6. Conclusions

The combination of FCM and QA is employed in this paper to optimize a network’s energy consumption and prolong the network’s lifetime. Initially, candidate CHs are selected based on a predefined energy threshold, prioritizing nodes with higher residual energy. Then, the proposed FQA considers effective parameters, including residual energy, the number of neighboring nodes, distance to the BS, and node centrality as descriptors of FIS to achieve the best decision, and the remaining nodes are assigned to particular CHs with higher potential according to energy and distance. Subsequently, an objective function is formulated based on potential energy derived from the energy matrix, distance matrix, and constraint terms. Then, we combine it with the kinetic energy generated by the interaction between n sets to obtain the Hamiltonian. The QA algorithm calculates the Hamiltonian to obtain the routing scheme from CHs to the BS with the minimum energy consumption. Finally, we utilized an on-demand re-clustering mechanism to decrease energy consumption further. Simulation results showed that FQA performs better than the existing protocols OAFS-IMFO, FC-RBAT, FRNSEER, and BOA-ACO in all scenarios regarding energy consumption, network lifetime, the number of surviving nodes, and throughput. Significantly, the network lifetime of FQA in scenario 1 has increased by 81.23%, 46.36%, 29.83%, and 14.95%, compared to OAFS-IMFO, FC-RBAT, FRNSEER, and BOA-ACO, respectively.

However, the proposed FQA is implemented only for a homogeneous and stationary network. In our future work, we will consider the incorporation of heterogeneous networks and mobile sinks into our research.

## Figures and Tables

**Table 1:** Input and output parameters.

Parameters	Linguistic Variables	Membership Functions
$f_{CH_{1}}$	VL (very low), VH (very high)	Trapezoidal
	L (low), M (medium), H (high)	Triangular
$f_{CH_{2}}$	F (few), N (numerous)	Trapezoidal
	M (medium)	Triangular
$f_{CH_{3}}$	N (near), F (far)	Trapezoidal
	M (medium)	Triangular
$f_{CH_{4}}$	L (low), H (high)	Trapezoidal
	M (medium)	Triangular
$CH_{probability}$	VP (very poor), VS (very strong)	Trapezoidal
	P (poor), $B_{ave}$ (below average), Ave (average),	Triangular
	$A_{ave}$ (above average), S (strong)	Triangular

**Table 2:** Optimized fuzzy rule table for CH selection.

No.	$f_{CH_{1}}$	$f_{CH_{2}}$	$f_{CH_{3}}$	$f_{CH_{4}}$	$CH_{probability}$
1	VL	F	N	L	$B_{ave}$
2	VL	F	M	L	$B_{ave}$
3	VL	F	F	L	P
4	VL	F	N	M	P
5	VL	F	M	M	P
6	VL	F	F	M	VP
⋮	⋮	⋮	⋮	⋮	⋮
73	M	N	N	L	S
74	M	N	M	L	$A_{ave}$
75	M	N	F	L	A
76	M	N	N	M	$A_{ave}$
77	M	N	M	M	A
78	M	N	F	M	$B_{ave}$
⋮	⋮	⋮	⋮	⋮	⋮
130	VH	N	N	M	VS
131	VH	N	M	M	VS
132	VH	N	F	M	$A_{ave}$
133	VH	N	N	H	S
134	VH	N	M	H	$A_{ave}$
135	VH	N	F	H	$A_{ave}$

**Table 3:** Simulation parameters.

Parameters	Value
Initial energy	1 J
$E_{elec}$	50 (nJ/bit)
$E_{pdb}$	5 (nJ/bit)
$\varepsilon_{fs}$	10 (pJ/bit/m^2^)
$\varepsilon_{mp}$	0.0013 (pJ/bit/m^4^)
$d_{0}$	87.7 m
Data packet size	4000 bits
Control packet size	200 bits
$M_{c}$	$10^{2}\sim 10^{6}$
Maxsteps	$10^{6}$
T	0.04
$\Gamma_{0}$	1.6

**Table 4:** Descriptions of scenarios.

Parameters	Scenario 1	Scenario 2	Scenario 3	Scenario 4
Area	100 m × 100 m	100 m × 100 m	200 m × 200 m	200 m × 200 m
Number of nodes	70	70	100	100
BS Location	x = 50, y = 50	x = 50, y = 100	x = 100, y = 100	x = 0, y = 0
Proportions of CHs	15%	15%	10%	10%

**Table 5:** FND, HND, LND in different scenarios.

Protocol		OAFS-IMFO	FC-RBAT	FRNSEER	BOA-ACO	FQA
Items						
	FND	539	785	739	803	997
Scenario 1	HND	1033	1244	1766	1288	1724
	LND	1566	1939	2186	2469	2838
	FND	340	807	876	760	324
Scenario 2	HND	1034	1209	1734	1294	1003
	LND	1343	1673	2145	2174	2489
	FND	280	629	518	104	362
Scenario 3	HND	551	941	854	1028	1418
	LND	1658	1775	2357	2635	3040
	FND	176	157	610	588	446
Scenario 4	HND	524	893	987	950	1030
	LND	1143	1249	1271	1307	1482

## Data Availability

Data are contained within the article.
